# The DEAD-box protein MEL-46 is required in the germ line of the nematode *Caenorhabditis elegans*

**DOI:** 10.1186/1471-213X-9-35

**Published:** 2009-06-17

**Authors:** Ryuji Minasaki, Alessandro Puoti, Adrian Streit

**Affiliations:** 1 Max Planck Institute for Developmental Biology, Spemannstrasse 35, D-72076 Tübingen, Germany; 2 Department of Biology, University of Fribourg, Chemin du Musée 10, CH-1700 Fribourg, Switzerland; 3 Max Planck Institute of Molecular Cell Biology and Genetics, Pfotenhauerstrasse 108, D-01307 Dresden, Germany

## Abstract

**Background:**

In the hermaphrodite of the nematode *Caenorhabditis elegans*, the first germ cells differentiate as sperm. Later the germ line switches to the production of oocytes. This process requires the activity of a genetic regulatory network that includes among others the *fem*, *fog *and *mog *genes. The function of some of these genes is germline specific while others also act in somatic tissues. DEAD box proteins have been shown to be involved in the control of gene expression at different steps such as transcription and pre-mRNA processing.

**Results:**

We show that the *Caenorhabditis elegans *gene *mel-46 *(maternal effect lethal) encodes a DEAD box protein that is related to the mammalian DDX20/Gemin3/DP103 genes. *mel-46 *is expressed throughout development and mutations in *mel-46 *display defects at multiple developmental stages. Here we focus on the role of *mel-46 *in the hermaphrodite germ line. *mel-46(yt5) *mutant hermaphrodites are partially penetrant sterile and fully penetrant maternal effect lethal. The germ line of mutants shows variable defects in oogenesis. Further, *mel-46(yt5) *suppresses the complete feminization caused by mutations in *fog-2 *and *fem-3*, two genes that are at the top and the center, respectively, of the genetic germline sex determining cascade, but not *fog-1 *that is at the bottom of this cascade.

**Conclusion:**

The *C. elegans *gene *mel-46 *encodes a DEAD box protein that is required maternally for early embryogenesis and zygotically for postembryonic development. In the germ line, it is required for proper oogenesis. Although it interacts genetically with genes of the germline sex determination machinery its primary function appears to be in oocyte differentiation rather than sex determination.

## Background

**Figure 1:**
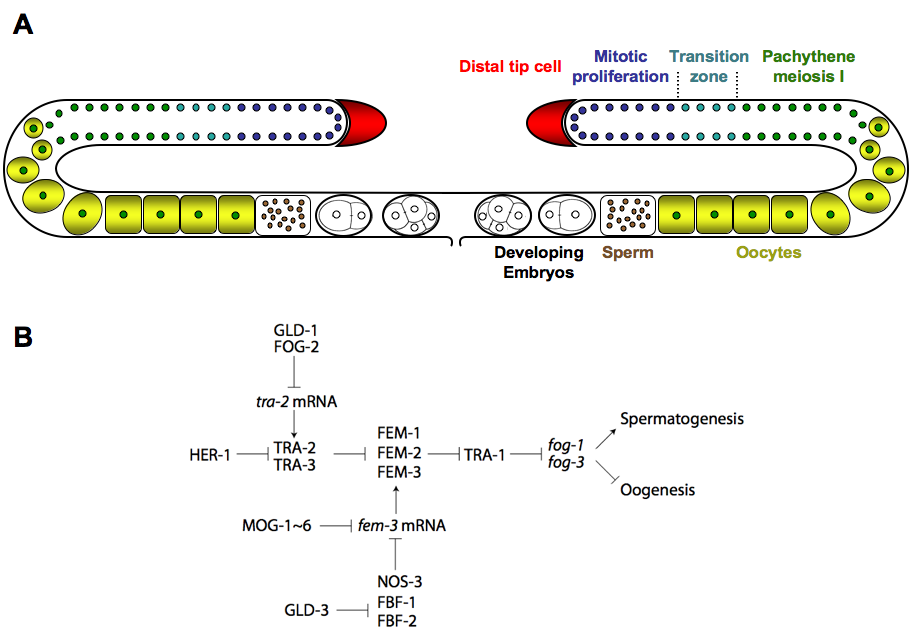
**The *C. elegans *gonad and germline sex determination**. (A) Schematic representation of the *C. elegans *adult hermaphrodite gonad and germ line. The gonad consists of two mirror imaged arms. The proximal ends and the vulva are on the ventral side and the syncycial distal portion is located dorsally. A signal from the somatic gonadal distal tip cell keeps the most distal germ line nuclei in mitosis. Nuclei that migrate towards the proximal part initiate meiosis, cellularize and differentiate into oocytes. The mature oocytes pass through the spermatheca where they are fertilized by sperm that had been produced earlier or that was deposited by a male. The embryos immediately initiate development and undergo the first few rounds of cell divisions in the uterus before they are deposited through the vulva. (B) Schematic and simplified genetic germline sex determination regulatory network. Arrows and T-bars represent positive and negative interactions, respectively. Capitalized gene names represent the proteins encoded by the respective genes.

The nematode *Caenorhabditis elegans *has two sexes, a self-fertile hermaphrodite and a male [1]. Hermaphrodites are somatically female and undergo a transient period of spermatogenesis during the L4 larval stage. Adult worms maintain the production of oocytes throughout the rest of their reproductive life. In the distal part of the gonad, a population of mitotically dividing cells is kept proliferative by a DELTA/NOTCH type signal that originates from the distal tip cell [2] (Fig 1A). Cells that migrate proximally initiate gametogenesis. The production of mature germ cells involves a switch from mitosis to meiosis and the initiation of either male or female differentiation [3]. To initially allow spermatogenesis to take place, the oogenesis promoting gene *tra-2 *is translationally repressed by the action of the RNA binding protein GLD-1 and the F box protein FOG-2 [4-6]. This leads to the activation of the three *fem *genes [7-9] and ultimately to the activation of the spermatogenesis promoting genes *fog-1 *and *fog-3 *[10,11] (Fig 1B). To switch to oogenesis, *fem-3 *is repressed post-transcriptionally by the concerted action of FBF-1, FBF-2 and NOS-3, which form a complex that binds to the 3' UTR of the *fem-3 *mRNA [12,13] and at least six *mog *genes [14,15]. As a consequence, *fog-1 *and *fog-3 *are repressed and oogenesis occurs. Strong loss-of-function (lf) mutations in any of the *fog *genes [16-18] or *fem *genes [7-9] and gain-of-function (gf) mutations in *tra-2 *[19] completely feminize the germ line and thereby transform the hermaphrodite into a female. Conversely *tra-2 *and *mog *lf mutants [14,15,19] and *fem-3 *gf mutants [20] fail to switch from spermatogenesis to oogenesis leading to a female soma with a gonad that is filled with sperm.

The exact mode of action of the six *mog *genes is not known. In addition to their function in germline sex determination, they are also maternally required for embryonic viability [14,15]. Three of the four *mog *genes that have been cloned so far encode DEAH box RNA helicases, namely, MOG-1, MOG-4 and MOG-5, which are the homologs of yeast splicing factors PRP16, PRP2, and PRP22, respectively [21,22]. Also *ddx-23*, which encodes a DEAD box protein homolgous to the yeast splicing factor PRP28 was found to be required for the sperm/oocyte switch [23].

DEAH and DEAD box proteins belong to the DExD/H RNA helicase family, which play many roles in RNA metabolism, for example, pre-mRNA splicing, ribosome biogenesis, RNA transport, translation initiation, and RNA decay [24-26]. Recently, the classical view that DExD/H proteins are exclusively involved in RNA metabolism was contested by evidence that several DExD/H proteins show non-RNA related activities that do not require their highly conserved helicase core motifs [27]. In *C. elegans*, several DEAD box RNA helicases are involved in germ line development. Loss of *glh-1 *and *glh-4 *(*G*erm *l*ine RNA *H*elicase) gene functions cause sterility due to failure in oogenesis and spermatogenesis [28,29]. Increased germline apoptosis was observed in the *cgh-1 *(*C*onserved *G*ermline *H*elicase) mutant [30]. *mut-14 *encodes yet another DEAD box helicase and is essential for germ line transposon inactivation and post-transcriptional gene silencing [31]. Other DEAD box RNA helicases are components of the P-granules [32], which are germ cell specific structures [33]. P-granules are considered to be pivotal for RNA metabolism as they contain many structural RNAs [34,35] and proteins involved in pre-mRNA splicing, RNA decay and translational activation [36-38]. The exact nature of how these DEAD box RNA helicases participate in the various features of germline development in *C. elegans *remains elusive.

In total, thirty-seven DEAD box and 11 DEAH box proteins are encoded in the *C. elegans *genome [39]. Systematic analysis of RNAi phenotypes suggested that more than half of these genes are essential and many are required for multiple developmental processes. However, for only a few of these genes has the characterization of mutants been reported. Here we describe a previously uncharacterized DEAD box protein-encoding gene, *mel-46*. Loss of *mel-46 *results in defects in several aspects of development. In this study, we particularly focus on the requirement for *mel-46 *in the germ line.

## Methods

### *Caenorhabditis elegans* strains and culture

General worm culturing and handling was done according to the standard methods [40,41].

The strain names and the full genotypes of the worm strains used were:

N2: Standard wild-type strain,

CB4856: polymorphic wild-type strain from Hawaii,

AH35: *unc-119(e2498)III; zhIs1[unc-119(+) *+ *lin-39::GFP]*,

AP32: *fem-3(q95gf)IV*,

BC1227: *sDf23/nT1 IV; +/nT1 V*,

CB1166: *dpy-4(e1166)IV*,

CB3844:*fem-3(e2006ts)IV*,

FR695: *sw1s15[ceh-13*(*enh740*)::*gfp *+ *rol-6*(*su1006)dm]V*,

JK509: *glp-1(q231)III*,

JK560:*fog-1(q253ts)I*,

JK574:*fog-2(q71)V*,

QA3: *egl-38*(*n578*) *mec-3*(*n3197*)*IV*; *sw1s15[ceh-13*(*enh740*)::*gfp *+ *rol-6*(*su1006)dm]V*,

QA243: *mel-46(yt5) IV; yDp1(IV;f)*,

QA269: *ytEx209[pRM8 (mel-46 rescue) + sur-5::gfp]; mel-46(yt5)IV*,

QA270: *ytEx210[pRM8 (mel-46 rescue) + sur-5::gfp]; mel-46(yt5)IV*,

QA273: *ytEx211[pRM8 (mel-46 rescue) + sur-5::gfp]; mel-46(tm1739)IV*,

QA298: *fem-3(e2006ts) mel-46(yt5)IV*,

QA300: *mel-46(yt5)/nT1IV; +/nT1V*,

QA310:*fog-1(q253ts)I; mel-46(yt5)IV*,

QA311:*mel-46(yt5)IV; fog-2(q71)V*,

RB1343: *T06A10.4(ok1475)IV*,

WH145: *cyk-2(oj34ts)IV*

### Molecular Biology

Standard techniques of molecular biology were performed as described [42].

### Microscopy

The specimens were prepared and analyzed following standard procedures [43]. For microscopy we used a Zeiss Axioplan 2 microscope fitted with a digital camera (MicroMax 5 MHz System, Princeton Instruments, Inc) or a Zeiss Axio Imager microscope with a SPOT camera (Diagnostic Instruments, Inc). Both microscopy setups were operated through the program MetaMorph (version 6.2r4 Universal Imaging Corporation™). The images were processed using either MetaMorph (version 6.2r4, Universal Imaging Corporation™) or Axiovision (release 4.2) and Adobe Photoshop (version 7.0).

### Isolation, backcrossing and balancing of the mutations *mel-46(yt5)* and *mel-46(tm1739)*

The mutation *mel-46(yt5ts) *was isolated in a screen for mutations that cause alterations in *ceh-13::gfp *(*enh740::gfp*) expression [44]. Prior to analysis the strain was backcrossed to N2 10 times. During this process the transgene *sw1s15[ceh-13*(*enh740*)::*gfp *+ *rol-6*(*su1006)dm]V *was removed from the strain. The mutation was balanced over *nT1 *(strain QA300), with the free duplication *yDp1 *(strain QA243) or the rescuing extrachromosomal arrays *ytEx209 *(strain QA269) or *ytEx210 *(QA270). Temporarily, the mutation was also maintained homozygously at permissive temperature between 15°C and 20°C. The deletion allele *mel-46(tm1739) *was generated by the National Bioresource Project for *Caenorhabditis elegans *in Japan upon our request and was sent to us by Dr S. Mitani. The mutation was backcrossed 10 times with N2 and balanced over the rescuing transgene *ytEx211 *resulting in strain QA273. This deletion starts at codon 173 of the predicted protein and removes part of exon 4 (41 bp), the entire intron 4 (65 bp) and exon 5 (125 bp) and part of intron 5 (194 bp).

### Sterility assay and dissection and DAPI staining of gonads

*mel-46(yt5)*/+ hermaphrodites were raised at 20°C and 25°C. Individual L1/L2 larvae were placed onto new plates and kept at the respective temperatures. Alternatively, progeny of *mel-46(yt5) *homozygous animals that had been raised at 15°C were picked and shifted to 25°C. After two days the plates were inspected. Animals that had not laid any embryos were considered sterile and animals that had laid all unhatched embryos were considered maternal effect lethal (Mel). Some of the adults were chosen randomly and their gonads dissected. The gonads were fixed in cold Methanol for 5 minutes at -20°C, transferred to PBS and DAPI (diamidinophenylindole) was added at a concentration of 1 μg/ml for 10 minutes.

### Determining gonad defects in sterile animals

Sterile non-transgenic animals of the strain QA269 were raised at 25°C and examined either by Nomarski DIC microscopy or by epi-illumination fluorescence after DAPI staining. 40× and 100× objectives were used for both types of microscopy.

### Brood size analysis

We placed progeny of *mel-46(yt5)*/+ mothers raised at either 15, 20, 23, or 25°C individually onto plates that were at the respective growth temperature. All animals were taken from cultures that had been maintained at their respective temperatures for at least two generations. Every day these animals were transferred to new plates and the embryos they produced were counted. Animals that produced no embryos were considered to be sterile, those that produced only or mainly dying embryos were considered to be Mel. Mel and sterile animals were considered to be *mel-46(yt5) *homozygotes. Worms that produced viable progeny were considered to be *mel-46(yt5)*/+ or +/+. The data for each temperature are the sum of three independent experiments.

### Construction and analysis of double mutant strains with *fem-3(e2006), fog-1(q253)* and *fog-2(q71)*

The double mutants were constructed as follows.

QA298: *fem-3(e2006ts) mel-46(yt5ts)IV*: We crossed CB3844:*fem-3(e2006ts) *hermaphrodites with *mel-46(yt5) *males at 15°C. The F1 progeny were cultured at 25°C and allowed to reproduce by self-fertilization. F2 animals were kept individually at 25°C until 24 h after they had laid the first embryos. Mel animals (*mel-46(yt5) *homozygotes) were shifted to 15°C in order to produce viable F3 animals. Later we lysed the F2 animals and searched for the recombinant genotype [*mel-46(yt5) fem-3(e2006)*/*mel-46(yt5) +*] by PCR and sequencing. The *mel-46(yt5) fem-3(e2006) *chromosome was made homozygous at 15°C in the following generations and the genotype confirmed. Note: the only molecular lesion we found in *fem-3(e2006) *was (AGT to AUA: Met_129 _to Ile) and not (AGT to AUA: Met_159 _to Ile) as originally reported [45]. We assume that it is due to a typing error in the original publication.

QA310: *fog-1(q253ts)I; mel-46(yt5ts)IV*: JK560:*fog-1(q253ts)I *hermaphrodites were crossed with *mel-46(yt5) *males at 15°C. The F1 generation was raised at 25°C and allowed to reproduce by self-fertilization. F2 animals were kept individually at 25°C until 24 hours after they had laid the first embryos. Mel animals (*mel-46(yt5) *homozygotes) were shifted to 15°C in order to produce viable F3 animals. F3 animals were picked individually to plates and double homozygous strains identified by PCR and sequencing of the molecular lesions.

QA311: *mel-46(yt5)IV; fog-2(q71)V*: JK574:*fog-2(q71) *females were crossed with *mel-46(yt5) *males at 15°C. The F1 animals were shifted to 25°C and F2 animals were raised at 25°C individually. Mel non Fog F2 animals were selected and shifted down to 15°C and allowed to reproduce by self-fertilization. Female F3 animals [*mel-46(yt5)/mel-46(yt5); fog-2(q71)*/*fog-2(q71)*] were crossed with *fog-2(q71) *males at 15°C. The resulting [*mel-46(yt5)/+; fog-2(q71)*/*fog-2(q71)*] males and females were crossed. In the next generation cultures were started with single pair crosses and double homozygous animals were identified by analysis of both molecular lesions.

#### Analysis of the mutant strains

A large number of the double mutants as well as the corresponding single mutant strains and the wild-type N2 strain were cultured at 15°C, then embryos were isolated by hypochloride treatment [41]. The embryos were cultured at 15°C for 12 hours before the hatched L1 larvae were transferred individually to plates and shifted to 25°C where they were incubated for 48 hours. We then searched for embryos that were present on the plates. Double mutants that produced embryos were kept and the total number of embryos they produced was determined. The data for each set (double mutant, single mutants, and wild type) are the sum of three independent experiments. In each experiment, all four strains were analyzed in parallel.

### Cloning of *mel-46*

We determined the approximate location of *mel-46(yt5) *at the right of linkage group IV by bulk segregant analysis single nucleotide polymorphism (SNP) mapping [46]. We then created strain *dpy-4*(*e1166*)*mel-46(yt5) *by interbreeding CB1166: *dpy-4(e1166) *and *mel-46(yt5) *and selecting double homozygotes in the F3 generation based on the Dpy and the Mel phenotypes (later this strain was unfortunately lost). We crossed *dpy-4*(*e1166*)*mel-46(yt5*) hermaphrodites with CB4856 males and allowed the progeny to reproduce by self-fertilization. In the next generation we selected Dpy non Mel animals for SNP analysis, which allowed us to place *mel-46(yt5) *very close to the right of a SNP (snp_Y43D4[1]) at the physical position 16728927. Direct sequencing of candidate genes from the *mel-46(yt5) *mutant genomic DNA revealed a premature stop (C168560033T, Q907Ochre) in the predicted gene T06A10.1. This mutation is neither present in the parental strain QA3 nor in the wild type strain N2. The small deletion allele *tm1739 *in T06A10.1 (see above) failed to complement *mel-46(yt5) *(see below). Further we were able to phenocopy the Mel-46(yt5) phenotype by RNAi (see below) and to rescue all aspects of the phenotypes of both mutations with a transgene where T06A10.1 was the only open reading frame (Plasmid pRM8 see below, strains QA269, QA270 and QA273).

### Complementation tests

#### *yt5* and *tm1739*

Wild type N2 hermaphrodites were crossed with QA158: *mel-46(yt5) *homozygous males, which had been growing at 15°C. The resulting males [*+*/*mel-46(yt5)*] were crossed with QA273: *ytEx211 *[pRM8:*mel-46*(+) + *sur-5::gfp*]; *mel-46*(*tm1739)IV *hermaphrodites. The SUR-5::GFP negative hermaphrodite progeny were picked at L1 stage and transferred to 25°C. The genotypes of these worms are expected to be 1/2 *+*/*mel-46(tm1739) *and 1/2 *mel-46*(*tm1739)*/*mel-46(yt5)*. Of the 63 animals analyzed 32 gave rise to viable progeny and we confirmed that their genotype was *+*/*mel-46(tm1739) *by PCR and by following the phenotypes in the next generation. The remaining 31 animals showed a fully penetrant Mel phenotype if they produced embryos and we confirmed that their genotype was *mel-46*(*tm1739)*/*mel-46(yt5)*] by PCR. This indicates that *yt5 *and *tm1739 *are allelic.

#### *yt5* and *ok1475*

RB1343: *T06A10.4(ok1475) *hermaphrodites were crossed with *mel-46(yt5) *homozygous males, which had been raised at 15°C. None of the F1 progeny showed a Mel phenotype (n > 30) at 25°C. We further confirmed the genotype by PCR. Therefore *yt5 *and *ok1475 *are not allelic.

#### *tm1739* and *ok1475*

QA273: *ytEx211 *[pRM8 (*mel-46 *rescue)+*sur-5::gfp*]; *mel-46*(*tm1739) IV *hermaphrodites were crossed with wild type males to obtain [*+*/*mel-46(tm1739)*]. The SUR-5::GFP negative males were then crossed with RB1343: *T06A10.4(ok1475)*. The genotypes of the cross progeny should be 1/2 [*+*/*T06A10.4(ok1475)*] and 1/2 [*mel-46*(*tm1739)*/*T06A10.4(ok1475)*]. We determined the genotype of 30 cross progeny by PCR and sequencing and found 14 to be [*+*/*T06A10.4(ok1475)*] and 16 to be [*mel-46*(*tm1739)*/*T06A10.4(ok1475)*]. All these animals grew normally and produced viable progeny. Therefore *tm1739 *and *ok1475 *are not allelic.

#### *yt5* and *sDf23*

BC1227: *sDf23/nT1 IV; +/nT1 V *hermaphrodites were crossed with the *mel-46(yt5) *homozygous males at 15°C. From the plates that had males we single-picked 80 F1 hermaphrodites and placed them at 25°C. None of these F1 hermaphrodites showed a Mel phenotype. As a control we followed the next generation of four randomly selected F1s and confirmed that *mel-46(yt5) *was present, showing that the original cross was efficient. This indicates that *sDf23 *does not uncover *yt5*.

#### *yt5* and *cyk-2(oj34)*

WH145: *cyk-2(oj34ts) IV *hermaphrodites were crossed with *mel-46(yt5) *males at 15°C. We picked many F1 progeny (n > 100) from the plates that had males and transferred them individually to new plates and shifted them to 25°C. The resulting F1 produced showed no Mel phenotype. This indicates that *yt5 *and *cyk-2(oj34ts) *are not allelic.

### Double stranded RNA interference (RNAi)

We purchased the T06A10.1 RNAi clone from Geneservice Ltd [47]. The clone contains 1135 base pairs of genomic sequence (physical position 16858332 to 16859466) flanked by T7 promoters. We amplified the T06A10.1 region by PCR with T7 promoter primers and used the product as template for *in vitro *transcription using the MEGAscript^® ^(Ambion) kit following the manufacturer's instructions. The resulting transcripts were heated to 95° for 5 minutes, then annealed at room temperature to produce double stranded RNA. The RNA was diluted in water to approximately 0.5 μg/μl and injected into the gonads of L4 or young adult hermaphrodites as described [48]. The injected worms were transferred to new plates 12 hours after injection and the progeny they produced was analyzed 24 to 36 hours after transfer.

### Transgenics

Extrachromosomal transgenes were obtained by micro-injection of DNA into the gonad of adult hermaphrodite worms [49]. The following concentrations were used to make extra-chromosomal arrays: *ytEx209 *and *ytEx210*, *ytEx211 *(pRM8 at 133 ng/μl and pTG96(*sur-5::GFP*) at 80 ng/μl) [50].

### Determination of the *mel-46* cDNA sequence

Total RNA was isolated from mixed staged worms using the Qiagen midi total RNA Kit (Cat. number 75142) according to the manufacturer's instructions. Reverse transcription was carried out with SuperScript™ II Reverse Transcriptase (Invitrogen) according to the manufacturer's instructions with random hexamer primers (for the 5' RACE) or primer BJ796 (5'-CCAGTGAGCAGAGTGACGAGGACTCGAGCTCAAGCT_15_V-3') (for the 3' RACE). One μl of the reverse transcription reaction was used as template for first round of PCR (30 cycles of 94° for 1 minute, 58° for 1 minute and 68° for 1 minute) using a primer against SL1 (for 5' RACE) or a primer that is complementary to BJ796 and different internal primers. One μl of the first round PCR was used as template for a second round of PCR with nested primers. A full list of all the primers used for the RACE experiments is available upon request. Two overlapping partial cDNA clones were generated. pRM3: a 1754 bp fragment extending from the splice leader SL1 to exon 8 was amplified using primers RM589-5'-GGTTCCTCTGTAAAATCGAATTTCGTC-3' and RS234-5'-GGTTTAATTACCCAAGTTTGAG-3' and cloned into TOPO pCR4 (Invitrogen). pRM5: a 2919 bp fragment ranging from exon 1 to the poly(A) tail was amplified using primers RM698- 5'-GAAAAATAGCCAATTCGACAGG-3' and BJ797 (see above) and cloned into TOPO pCR4 clones (Invitrogen). The full-length cDNA clone pRM7 was created by replacing the *Not*I (in the pCR4 vector) – *Sal*I (in exon 5) fragment of pRM5 with the *Not*I – *Sal*I fragment of pRM3. The full-length *mel-46 *cDNA is 2922 bp.

### GenBank accession number

The accession number for the full-length *mel-46 *cDNA sequence is [GenBank:EU051652].

### Making of *mel-46* rescue constructs

The *Not*I (in the vector) – *Sal*I (in exon 5) fragment of pRM7, the clone that contains the full length *mel-46 *cDNA, was replaced with the genomic *Not*I-*Sal*I fragment that extends from 1759 bp upstream of the translational start codon to exon 5. The genomic fragment was created by PCR amplification with primers RM1033-TATAGCGGCCGCTGCATGTGAAGTGGAACCAT and RM1031-ACCAGCGTTAAACCGAACAA followed by the restriction digestion with *Not*I and *Sal*I. The resulting plasmid pRM8 hence consists of 1759 bp promoter, exons and introns 1 – 4, exons 5 – 10 and the *mel-46 *3'UTR in the backbone of TOPO pCR4.

### Anti-MEL-46 antibody and Western Blotting

A Polyclonal anti-MEL-46 antibody was raised against the peptide CPFEERLRRQRKREK (AA_724–739_) and affinity purified by Biogenes, Berlin. For Western blotting, approximately 100 μl of worms were harvested and dissolved in 500 μl of sample buffer (Tris-HCl 60 mM, 25% Glycerol, 2% SDS, 14.4 mM Beta-marcaptoethanol) by boiling for five minutes. After adjusting the approximate concentration of the protein samples, 5–10 μl per lane were loaded onto standard SDS-PAGE gels. These gels were blotted onto Hybond-P Nylon membranes (Amersham Bioscience) according to the manufacturers' instructions. For detection the anti-MEL-46 antibodies were used at 1:2500. For the loading control, a polyclonal anti-alpha-tubulin antibody (Dianova, Hamburg) was used at 1:2500. As secondary antibody, we used an alkaline phosphatase conjugated goat anti-rabbit antibody from Dianova (Hamburg) at 1:1000 dilution and we detected the signals using the premixed PCIP/NBT solution (Sigma) according to manufacturer's instructions.

## Results

### Isolation of the mutation *yt5*

**Figure 2:**
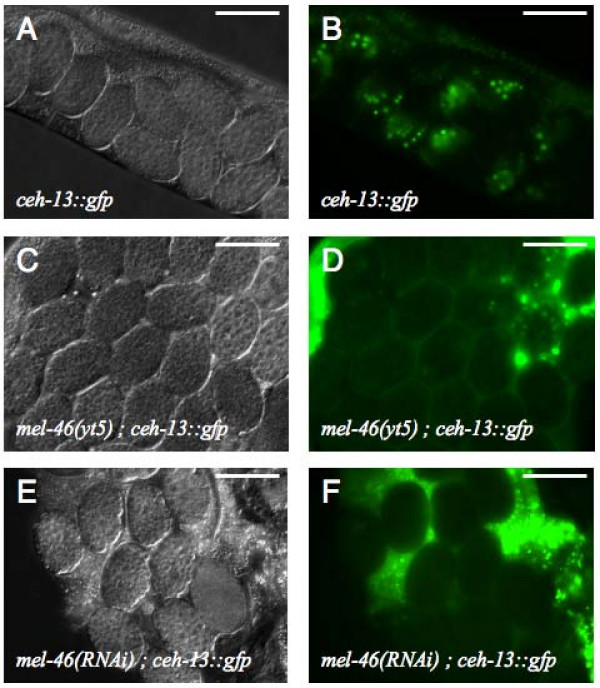
***mel-46(yt5) *is maternal effect lethal and fails to express *ceh-13::gfp***. Genotypes and reporter constructs are indicated. (A, C, E) DIC images of live animals. (B, D, F) fluorescent pictures of the same embryos shown in A, C, and E respectively. Compared to B, panels D and F were deliberately over exposed. Therefore the background caused by autofluorescence of the intestine appears stronger. All images were taken with a 40× objective. The scale bars are 50 μm.

The *yt5 *allele was originally isolated in a genetic screen for mutations that abolish or alter the expression of an early embryonic *ceh-13::gfp *reporter construct (Fig 2B) [44]. At 25°C *yt5 *homozygous hermaphrodites displayed various defects in the germ line (see below) and those animals that were not sterile showed a fully penetrant maternal effect lethal (Mel) phenotype. Based on this phenotype, the gene genetically defined by *yt5 *was named *mel-46*. *mel-46(yt5) *is not a null allele. However, the facts that it is fully recessive and that *mel-46 *RNAi experiments lead to a fully penetrant Mel phenotype and loss of *ceh-13::gfp *expression (Fig 2F) suggest that *yt5 *is a loss-of-function allele (see discussion). Embryos that are the progeny of *mel-46(yt5) *homozygous mothers invariably die with about 100 cells without any sign of morphogenesis and fail to express *ceh-13::gfp *(Fig 2D). At 20°C and below, the Mel phenotype is incompletely but still highly (>90%) penetrant while most embryos express the *ceh-13::gfp *marker normally (data not shown). *mel-46(yt5) *males can sire a considerable number of progeny at 15°C. At 25°C they appear to be sterile although they look morphologically wild type. The details of the phenotype in the embryos will be described elsewhere and this publication concentrates on the cloning of *mel-46 *and its requirement in the germ line.

### *mel-46* encodes a putative DEAD box RNA helicase

**Figure 3:**
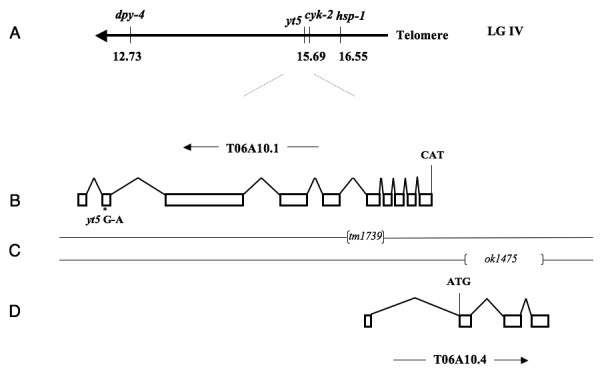
**Structure of the *mel-46 *locus**. (A) Schematic representation of the genetic map of the right end of chromosome IV. (B) Structure of the predicted gene T06A10.1. The direction of transcription (arrow), the translation start codon (CAT) and the position of the G to A transition in *yt5 *are indicated. (C) position of the two small deletions. The regions between brackets are deleted in the corresponding deletion mutants. (D) The predicted structure of T06A10.4, the translation start codon (ATG) and the direction of transcription (arrow) are indicated. *ok1475 *fully complements *tm1739 *and *yt5*.

**Figure 4:**
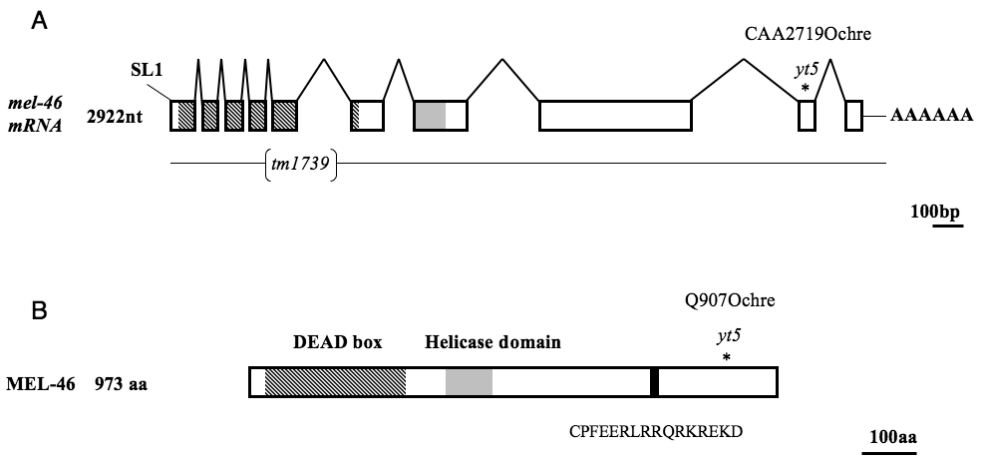
**The *mel-46 *gene and protein structure**. (A) *mel-46 *mRNA. The 10 exons are represented by boxes with lines (introns) joining them. The *mel-46 *mRNA is trans-spliced to the spliced leader SL1. The asterisk designates mutation *yt5 *(C → T) that leads to a premature stop codon at position 2719. The deletion allele *tm1739 *removes 425 bp of the genomic sequence. The coding regions for the DEAD box and the predicted helicase domain are shown in shaded and gray, respectively. (B) MEL-46 protein. The mutation *yt5 *creates a truncated protein. The DEAD box (shaded box), the helicase domain (grey box) and the 16 amino acid oligo-peptide that was used to raise antibodies (black box) are indicated.

Using a combination of classical two-point mapping and SNP mapping we mapped the mutation *yt5 *to position 15.69 on the right arm of chromosome IV (Fig 3A). *yt5 *complemented the maternal effect lethal mutation *cyk-2(oj34ts) *and the deficiency *sDf23*, both of which reside in this region. We identified a point mutation in the predicted gene T06A10.1 of *yt5 *homozygotes that introduced a premature translational stop codon towards the end of exon 9 (Fig 3B and 4A). Further, we received a small deletion allele (*tm1739*) that removes 425 bp and is likely to be a full knock out (Fig 3C and 4A). *tm1739 *failed to complement *yt5*. However, *tm1739 *homozygous animals arrest as L4 larvae. This indicates that there is a zygotic function of *mel-46 *that can be fulfilled by the *yt5 *mutant form of MEL-46. The upstream part of *mel-46 *overlaps with a non-coding exon of another predicted gene, T06A10.4. This predicted gene is transcribed in the opposite direction (Fig 3D). To exclude the possibility that this gene was responsible for some of the effects we observed, we requested the deletion allele *ok1475 *that takes out most of the coding region of T06A10.4 from exon 1 to exon 3 (Fig 3C). The deletion is 969 bp upstream of the *mel-46 *translation start codon at the physical position 16864367 to 16865184. *ok1475 *is homozygous viable and fully complemented *mel-46(yt5) *and *mel-46(tm1739)*. Further, a transgene that contained all of T06A10.1, but not the entire T06A10.4, rescued the *yt5 *and *tm1739 *mutations and RNAi with T06A10.1 phenocopied *mel-46(yt5) *(Fig 2E, F). From these results we conclude that T06A10.1 is *mel-46*. We determined the gene structure by analyzing the *mel-46 *cDNA (Fig 4A). *mel-46 *encodes a 973 amino acid protein that contains a DEAD box and a helicase domain (Fig 4B). MEL-46 and human Ddx20/Gemin3/DP103 are reciprocal best BLAST hits, indicating that these two proteins are related. The similarity is, however, limited to the N-terminal half of the protein that contains the DEAD box and the helicase domain.

### MEL-46 is expressed throughout development

**Figure 5:**
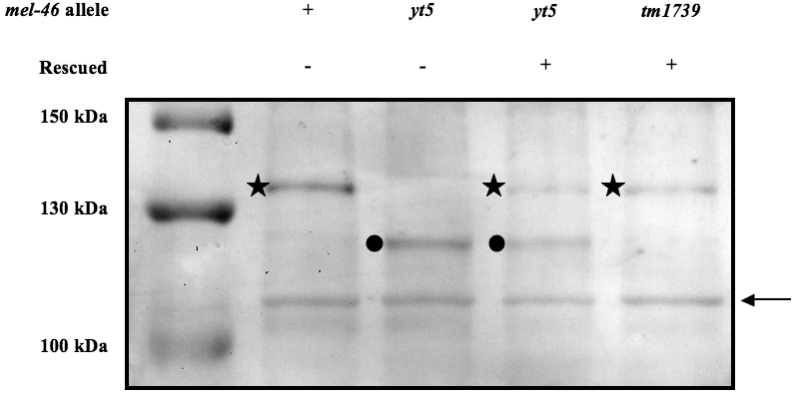
**The anti-MEL-46 antibody is specific and the truncated protein is produced and stable in *mel-46(yt5) *mutants**. Western blot analysis of protein extracts from wild type, *mel-46(yt5)*, *mel-46(yt5) *rescued with a wild type copy expressed from a transgene and *mel-46(tm1739) *rescued with a wild type copy expressed from a transgene. All extracts are from worms that were raised at 25°C. Asterisks designate a band that is specific for worms that contain a wild type copy of *mel-46*, filled circles point to a band that is specific for worms with a *mel-46(yt5) *mutant copy. Both bands appear somewhat larger than predicted based on the protein sequence (110.4 kD and 103.1 kD respectively). The band around 110 kDa that is present in all lanes (arrow) is also detected by the pre-immune serum (data not shown) and serves here as an internal loading control.

*In situ *hybridization images available from the Nematode Expression Pattern Database (; clone 90g5, accession numbers D74979 and D72164) show a strong signal in the gonad of the adult and in young embryos, which indicates that the RNA is present at these stages. Quantitative RT-PCR experiments confirmed this finding but also showed that the RNA is not restricted to adults and embryos and is also in the somatic tissues of adults (data not shown). To determine when and where the MEL-46 protein is present, we raised an antibody against an oligo-peptide (Fig 4B). On western blots the antibody recognizes a band above the 130 kD marker in extracts from wild type (N2) worms (Fig 5). In *mel-46(yt5) *mutants there is a novel band below the 130 kD marker. In extracts from worms that were homozygous for *mel-46(yt5) *and carried a wild type copy of the gene on a rescuing transgene, both bands are visible. Finally, in worms that are homozygous for the putative null allele *mel-46(tm1739) *and rescued by a transgene, only the wild type band is visible. This shows that the antibody recognizes MEL-46 and that the truncated protein is present in the *yt5 *mutants.

**Figure 6:**
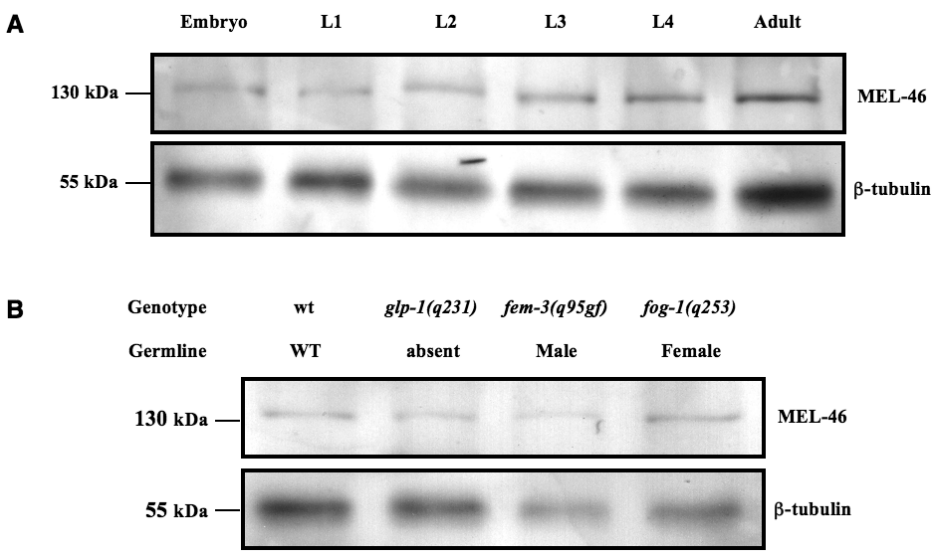
**MEL-46 expression**. (A) MEL-46 is expressed throughout the life cycle. Western blot with extracts from wild type worms of the indicated developmental stages, probed with anti-MEL-46 (top) and anti-β-tubulin (bottom). (B) MEL-46 is not restricted to the germ line in the adult. At 25°C, *glp-1(q231) *animals have no germ line, *fem-3(q95gf) *animals are somatically hermaphrodite but they only produce sperm, *fog-1(q253) *animals make only oocytes and no sperm.

Consistent with the pleiotropic phenotype of the mutation, the MEL-46 protein is present at all developmental stages (Fig 6A) and is not limited to the hermaphrodite germ line in adults since it is also found in hermaphrodites that lack a germ line or have a germ line that is either masculinized or feminized (Fig 6B).

### *mel-46(yt5)* is a partially penetrant temperature sensitive sterile allele

At 25°C about one third of the *mel-46(yt5) *homozygous hermaphrodites that were the progeny of heterozygous mothers were sterile, meaning they did not produce any embryos (Table 1). The penetrance of the sterility was somewhat higher in animals that were the progeny of homozygous mothers and were shifted from 15°C to 25°C at the L1 stage (Table 1). This effect is fully temperature sensitive since we did not observe any sterile worms at 20°C (Table 1). In order to further analyze the germline defects, we dissected gonads from *mel-46(yt5) *hermaphrodites and their non-mutant siblings and stained them with DAPI. The mutants were picked randomly without paying attention if they were sterile or Mel. Among 77 gonads analyzed from *mel-46(yt5) *animals that were raised at 25°C, 42 (54.5%) showed obvious defects (2, and see below). The manifestation of these defects was temperature dependent because of the 71 gonads from *mel-46(yt5) *animals that were raised at 20°C, all looked superficially normal. In the following we analyzed sterile and fertile animals separately.

**Table 1:** Temperature dependent sterility of *mel-46(yt5) *hermaphrodites

Genotype mother	Temperature	n	WT (%)^c^	Mel (%)^d^	Sterile (%)^e^	Sterile among mutant (%)^f^
*mel-46(yt5)*/+	20°C^a^	240	177 (73.8)	63 (26.3)	0	0
*mel-46(yt5)*/+	25°C^a^	622	460 (74)	110 (17.7)	52 (8.4)	32.10
*mel-46(yt5)/mel-46(yt5*)	25°C^b^	1299	0	674 (51.9)	625 (48.1)	48.33

^a ^The mothers and the analyzed progeny were permanently kept at the indicated temperature. ^b ^the analyzed worms where shifted to the temperature indicated as L1 larvae. ^c ^Phenotypically wild type, these worms were considered to be *mel-46(yt5)*/+ or +/+.^d ^Maternal effect lethal, these worms produced embryos, most (at 20°C) or all (at 25°C) of which arrested with less than 100 cells (see Fig.1). ^e ^These worms did not produce any embryos. ^f ^Mel and Sterile worms were considered to be mutant.

**Table 2:** Temperature dependent germline defects in *mel-46(yt5) *mutants

Genotype^a^	Temperature	Germline defect^b^	n^c^	%
+/+ or *mel-46(yt5)*/ +	20°C	0	42	0.0
*mel-46(yt5)*		0	71	0.0

+/+ or *mel-46(yt5)*/ +	25°C	0	31	0.0
*mel-46(yt5)*		42	77	54.5

^a ^All animals were the progeny of *mel-46(yt5)*/ + mothers. Animals that produced predominantly (at 20°C) or exclusively (at 25°C) dying embryos were considered to be *mel-46(yt5) *homozygous. ^b ^Number of gonads with any of the defects described in the text. ^c ^Number of gonads analyzed.

**Figure 7:**
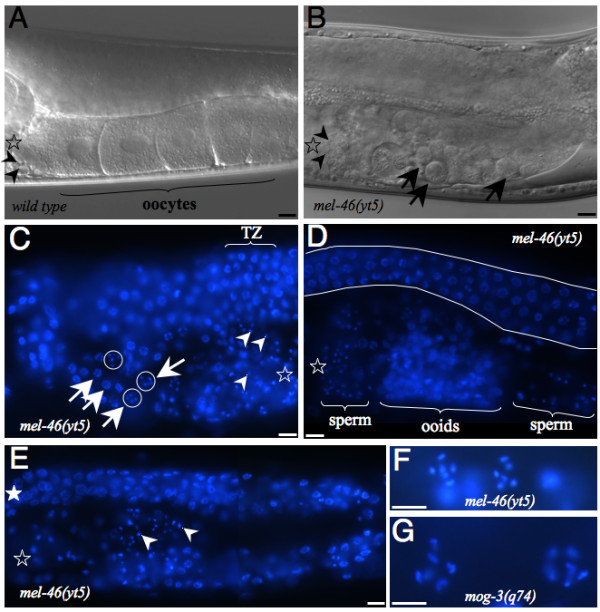
***mel-46(yt5) *mutation causes variable germline defects at 25°C**. (A) DIC image of a wild type animal. Arrowheads point to spermatids. (B) DIC image of a *mel-46(yt5) *homozygous animal. Arrowheads point to sperm cells, arrows point to ooids. (C) *mel-46(yt5) *homozygous animal of class 1 (see text) stained with DAPI. Arrowheads point to sperm nuclei. Arrows point to ooid nuclei in meiotic prophase, ooid nuclei in diakinesis are circled. TZ: transition zone, the mitotic region is on the left, meiotic pachytene is on the right of TZ. D: *mel-46(yt5) *homozygous animal of class 2 (see text). The visible portion of the distal arm is seen between the two white strokes. (E) A whole gonad arm from a class 3 mutant stained with DAPI. Detail of two nuclei in diakinesis from a *mel-46(yt5) *mutant of class 1 (F) and from a *mog-3(q74) *animal that has produced a few oocytes (G). The six pairs of chromosomes are made visible by DAPI. Open stars designate the proximal end of the germ line, the filled star indicates the distal end. The scale bar represents 10 μm in all panels. Images in panels A to F to were taken with a 40× objective, and those in panels G and H were taken with a 100× objective.

To get an idea of what might cause the sterility we inspected the gonads of sterile *mel-46(yt5) *animals more closely. Among 57 gonad arms analyzed, none contained normal, mature oocytes and we could divide them into three classes of defects: Class 1: the germ lines failed to produce mature oocytes. Instead, smaller oocyte-like cells, also called ooids, were generated (Compare Fig 7A to Fig 7B, C). However, the overall organization of the germ line was normal. The number of ooids varied from 0 to 20 ooids or more per gonadal arm. Occasionally, ooid nuclei had entered diakinesis (Fig 7C, circled and Fig 7F). The amount of sperm was variable as well, ranging from less than 40 to 350 cells per gonad arm, the average being 121. Fifty-one (89.5%) of the 57 gonadal arms fell into this class.

Class 2: a more severely disorganized arrangement of germ nuclei. Sperm, spermatocytes and ooids were mixed within the same region of the gonad (Fig 7D). Typically ooid nuclei had not entered diakinesis. Four (7%) of the 57 gonadal arms belonged to this class.

Class 3: worms with gonads that were much smaller than normal. Gonadal arms were thinner and contained less than 130 nuclei in mitosis or in meiotic prophase. Neither oocytes nor ooids have been observed in these germ lines (Fig 7E). Two (3.5%) of the 57 gonadal arms belonged to this class.

The animals that were not sterile showed a temperature dependent reduction in the number of embryos they produced, here referred to as brood size (Table 3). While the brood sizes of *mel-46(yt5) *mothers were not significantly different from their non-mutant siblings at 15°C and at 20°C, their brood sizes were clearly reduced at 23°C (210.2 ± 66.73 versus 277.6 ± 41.3, p = 0.006) and at 25°C (45.4 ± 32.9 versus 185.0 ± 23.7, p = 2.0^e-10^).

**Table 3:** Temperature dependent decrease in brood size of *mel-46(yt5) *mutants

Temperature	Genotype^a^	n^b^	Mean	Std Dev	p^c^
15°C	+/+ or *mel-46(yt5)*/ +	12	306.5	20.8	0.492
	*mel-46(yt5)*	13	294.6	36.2	

20°C	+/+ or *mel-46(yt5)*/ +	20	309.4	29.7	0.743
	*mel-46(yt5)*	10	305.2	54.5	

23°C	+/+ or *mel-46(yt5)*/ +	14	277.6	41.3	0.00644
	*mel-46(yt5)*	14	210.2	66.7	

25°C	+/+ or *mel-46(yt5)*/ +	20	185.0	23.7	1.99^e-10^
	*mel-46(yt5)*	17	45.4	32.9	

^a ^All animals were the progeny of *mel-46(yt5) */ + mothers. Animals that produced predominantly (at lower temperatures) or exclusively dying embryos were considered to be *mel-46(yt5) *homozygous. ^b ^Only animals that were fertile were included in this analysis. ^c ^two tailed paired t-test between the genotypes within the temperature.

### *mel-46(yt5)* weakly suppresses some mutations that cause germline feminization

As described above, in many *mel-46(yt5) *gonads germ cells failed to differentiate as oocytes. However, all worms made sperm and some even showed an elevated number of sperm, which lead us to suspect that *mel-46(yt5) *leads to a slight masculinization of the germ line. To test this hypothesis we asked if *mel-46(yt5) *can suppress mutations that feminize the germ line. The mutations *fog-1(q253), fog-2(q71) *and *fem-3(e2006) *are known to completely feminize the germ line leading to complete self-sterility [7,18,51] and are at the bottom (*fog-1*), the top (*fog-2*) or in the middle (*fem-3*) of the genetic cascade that determines germline sex [52] (Fig 1B). We constructed double mutant strains for *mel-46(yt5) *with these three mutations and asked if the double mutants would produce embryos, indicating that they made functional sperm. We found that *mel-46(yt5) *was capable of weakly suppressing *fog-2(q71) *and *fem-3(e2006) *but not *fog-1(q253) *(Table 4). Sixteen (3.5%) out of 461 *mel-46(yt5); fog-2(q71) *gave rise to an average of 24 embryos per hermaphrodite while none of the *fog-2(q71) *single mutants produced any embryos (n = 457). Eighteen (3%) out of 594 *fem-3(e2006) mel-46(yt5) *produced an average of 9 embryos per animal while none of the *fem-3(e2006) *worms produced embryos (n = 416). All embryos produced by either of the two double mutants arrested with a phenotype indistinguishable from *mel-46(yt5) *mutants. On the other hand, neither *fog-1(q253) *single mutant (n = 739) nor *fog-1(q253); mel-46(yt5) *(n = 610) animals produced any embryos. In spite of the caveat that the mutant alleles used in our analyses are not null (see discussion), altogether, our results suggest that *mel-46(yt5) *masculinizes the germ line and acts downstream of or in parallel with *fem-3 *and *fog-2 *and our results are consistent with a role of *mel-46 *upstream of *fog-1*.

**Table 4:** Genetic interaction of *mel-46 *with *fog-1*, *fem-3*, and *fog-2*

Genotype	WT^a^	Mel^b^	Sterile^c^	n
wt	480 (98.4)	0 (0)	8 (1.6)	488
*mel-46(yt5)*	0 (0)	265 (55.3)	214 (44.7)	479
*fog-1(q253)*	0 (0)	0 (0)	739 (100)	739
*fog-1(q253); mel-46(yt5)*	0 (0)	0 (0)	610 (100)	610

wt	328 (99.1)	0 (0)	3 (0.9)	331
*mel-46(yt5)*	0 (0)	201 (62.6)	120 (37.4)	321
*fem-3(e2006)*	0 (0)	0 (0)	416 (100)	416
*fem-3(e2006) mel-46(yt5)*	0 (0)	18 (3)	576 (97)	594

wt	328 (99.1)	0 (0)	3 (0.9)	331
*mel-46(yt5)*	0 (0)	163 (39.5)	250 (60.5)	413
*fog-2(q71)*	0 (0)	0 (0)	457 (100)	457
*mel-46(yt5); fog-2(q71)*	0 (0)	16 (3.5)	445 (96.5)	461

^a^Individuals that produced live progeny. ^b ^Individuals that produced dying embryos (see Fig. 1).^c ^Animals that did not produce any embryos. Values in the brackets are the percentage of the total animals analyzed. All experiments were done at 25°C.

## Discussion

Here we report the genetic isolation, molecular cloning and an initial phenotypic analysis of the gene *mel-46*. This gene encodes a protein of the DEAD box family and is required for embryogenesis, larval development and proper germline differentiation. Interestingly, also the mouse and the fly homologs of *mel-46 *were recently shown to be required for development and for proper function of the germ line. Mouillet et al. (2008) [53] demonstrated that murine DP103/Ddx20 is essential for early embryogenesis and influences ovarian morphology and function. Mutations in the *Drosophila melanogaster *Ddx20 ortholog, *gemin3*, lead to larval arrest and defects in the nurse cells in the germ line[54].

In *C. elegans*, the mutant phenotype of *mel-46 *is very reminiscent of the phenotype of *ddx-23*, a gene that also encodes a DEAD box protein. *ddx-23 *is homologous to human DDX23 and the yeast splicing factor PRP28. Analysis of mutants and RNAi knock down demonstrated that this gene is also required for embryonic viability, larval development and proper germline growth and differentiation, including the sperm-oocyte switch [23].

It is quite common that DEAD/H box proteins play multiple roles, both genetically and biochemically. A systematic RNAi analysis of genes that encode Helicase like proteins suggested that many DEAD/H box protein encoding genes are required multiple times during *C. elegans *development [39]. Many proteins of this family possess RNA helicase activity and use ATP hydrolysis as an energy source to unwind RNA structures or separate RNA-protein complexes [24-26]. However, several members of this family are also involved in transcriptional regulation [27]. For example the mammalian Ddx20/Gemin3/DP103 protein has been independently isolated as a transcriptional regulator of viral and nuclear genes (as Ddx20/DP103) [55-58], and as a component of the nuclear and cytoplasmic SMN complex (as Gemin3) [59], which plays roles in snRNP metabolism.

### *mel-46* alleles

We describe two different mutant alleles of *mel-46*. The first one, *yt5*, is the reference allele and its molecular lesion is a premature stop codon towards the end of the penultimate exon. The second allele, *tm1739*, is a small deletion that takes out the entire exon 5 and would introduce a translational frame shift in the conserved DEAD box if exon 4 were spliced to exon 6. This mutation is therefore very likely to be a null. *yt5 *is temperature sensitive, and even at the restrictive temperature of 25°C, it is not null. The knockout allele *tm1739 *causes larval lethality that we did not observe in *yt5 *homozygous and *yt5/tm1739 *trans-heterozygous animals. Consistent with this idea, a truncated MEL-46 protein is present in *mel-46(yt5) *mutants (Fig 5) and might still perform some of the functions. The fact that *yt5 *is temperature sensitive and the *yt5 *mutant animals develop to adulthood allowed us to study the function of *mel-46 *in the germ line. However, we cannot completely exclude a neomorphic contribution to the phenotype that is caused by the truncated protein. However the *yt5 *mutation is fully recessive and *mel-46 *RNAi experiments lead to a fully penetrant Mel phenotype that was indistinguishable from that of *yt5*, indicating that *yt5 *is essentially a loss-of-function allele.

### Germline defects in *mel-46(yt5)*

At 25°C *mel-46(yt5) *mutants display a range of germline defects. Some of the *mel-46(yt5) *adults produce embryos that undergo several rounds of cell division, indicating that these animals make functional gametes of both sexes. However, they have smaller broods than wild type animals demonstrating that their germ line is not entirely normal. Other hermaphrodites were defective in the proper formation of oocytes and did not produce any embryos. All animals did form normal looking sperm. In addition, *mel-46(yt5) *can partially suppress the complete self-sterility of the *fog-2(q71) *and *fem-3(e2006) *but not *fog-1(q253)*. Although the suppression was weak it has to be taken into consideration that we were asking for suppression of feminizing mutations to the point that the double mutants produced functional sperm and functional oocytes. Being so rigorous it is very likely that we underestimate the masculinizing effect of *mel-46(yt5)*. Our results are consistent with a genetic requirement of *mel-46 *for female germ cell differentiation downstream or in parallel of *fog-2 *and *fem-3 *and upstream of *fog-1*. However, although all mutant alleles used in this study are fully penetrant at 25°C, it has to be noticed that they are probably not null. For this reason and because the sex determining pathway is quite complex, the epistatic relationship of *mel-46 *with these three genes cannot be definitely determined with our assays. Although *mel-46(yt5) *clearly leads to a partial masculinization of the germ line, at the moment we do not know if this mutation affects sex determination specifically or if it also influences the function of other genes. The Mel-46 phenotype cannot be considered a classical Mog phenotype for various reasons. First, Mog animals produce large amounts of sperm (400 to 800 sperms per ovotestis [14]). We observed only a small fraction of animals with a slightly elevated sperm count and the average number of sperm was normal. Second, in the few *mog *mutants that occasionally produce limited amounts of oocytes when grown at permissive temperature, the spermatogenic region is distinct from that of oogenesis [14]. This is different in *mel-46(yt5) *animals, as ooids were sometimes mixed with sperm. Third, when Mog animals are maintained to produce oocytes or ooids (for example in the case of a temperature downshift for *mog-3(q74)*), oocyte nuclei enter diakinesis with their chromosomes well separated (Fig 7G). In contrast in *mel-46(yt5) *mutants of class 1, only a small subset of ooids entered diakinesis. Moreover, such nuclei are significantly smaller as compared to those in *mog-3(q74) *animals (Fig 7F). Therefore, we suggest that the Mel-46 phenotype is primarily a defect in oocyte differentiation.

## Conclusion

Here we report the genetic isolation, cloning and molecular and phenotypic characterization of the *C. elegans *gene *mel-46*. This gene encodes a putative DEAD box helicase and is required at least three times during development, namely: i) maternally for embryogenesis; ii) zygotically for progression through larval development and iii) in the hermaphrodite germ line. Consistent with these observations, we found the MEL-46 protein to be present at all developmental stages and in the germ line and in the soma. In this publication we focus on the requirement of *mel-46 *in the hermaphrodite germ line. We demonstrate that *mel-46 *weakly suppresses the feminizing mutations *fog-2(q71) *and *fem-3(e2006) *but not *fog-1(q253)*. Although this genetic interaction indicates a certain feminizing activity of *mel-46 *in the germ line, we speculate that its primary function is in oocyte differentiation rather than sex determination.

## Authors' contributions

RM did most of the bench work. He was involved in the experimental design, the analysis and interpretation of the data and in writing of the manuscript. AP took part in the design, the execution and the interpretation of the analysis of the germ line defects. He was also involved writing the manuscript. AS supervised the project. He was involved in the experimental design and in the analysis and interpretation of the data. He did some of the bench work and wrote the manuscript with the assistance of RM and AP. All authors read and approved the final manuscript.
